# Prescreening and treatment of aortic dissection through an analysis of infinite-dimension data

**DOI:** 10.1186/s13040-021-00249-8

**Published:** 2021-04-01

**Authors:** Peng Qiu, Yixuan Li, Kai Liu, Jinbao Qin, Kaichuang Ye, Tao Chen, Xinwu Lu

**Affiliations:** 1 Department of Vascular Surgery, Shanghai Ninth People’s Hospital Affiliated with Shanghai Jiao Tong University School of Medicine, Shanghai, China; 2 Big Data Research Lab, University of Waterloo, Waterloo, Canada; 3 Department of Economics, University of Waterloo, Waterloo, Canada; 4 Stoppingtime (Shanghai) BigData & Technology Co. Ltd., Shanghai, China; 5 School of Mathematical and Computational Sciences, University of Prince Edward Island, Charlottetown, Canada; 6 Senior Research Fellow of Labor and Worklife Program, Harvard University, Cambridge, USA

**Keywords:** Aortic dissection, Pre-disease screening, In-hospital treatment, Image recognition, Functional data analysis

## Abstract

**Background:**

Aortic dissection (AD) is one of the most catastrophic aortic diseases associated with a high mortality rate. In contrast to the advances in most cardiovascular diseases, both the incidence and in-hospital mortality rate of AD have experienced deviant increases over the past 20 years, highlighting the need for fresh prospects on the prescreening and in-hospital treatment strategies.

**Methods:**

Through two cross-sectional studies, we adopt image recognition techniques to identify pre-disease aortic morphology for prior diagnoses; assuming that AD has occurred, we employ functional data analysis to determine the optimal timing for BP and HR interventions to offer the highest possible survival rate.

**Results:**

Compared with the healthy control group, the aortic centerline is significantly more slumped for the AD group. Further, controlling patients’ blood pressure and heart rate according to the likelihood of adverse events can offer the highest possible survival probability.

**Conclusions:**

*The degree of slumpness* is introduced to depict aortic morphological changes comprehensively. The morphology-based prediction model is associated with an improvement in the predictive accuracy of the prescreening of AD. The dynamic model reveals that blood pressure and heart rate variations have a strong predictive power for adverse events, confirming this model’s ability to improve AD management.

**Supplementary Information:**

The online version contains supplementary material available at (10.1186/s13040-021-00249-8).

## Background

Among all aortic diseases, aortic dissection (AD) is one of the most catastrophic and is associated with a high mortality rate [1–5]. In contrast to the drastic advances for most cardiovascular diseases [6, 7], both the incidence and in-hospital mortality rate of AD have increased over the past 20 years: the incidence has risen from 4.4 to 5.3 per 100 000 person-years from 1995 to 2015 [8], and the mortality rate has risen from 12% to 14% from 1995 to 2013 [9]. There are three main reasons for this stagnation. First, the asymptomatic nature of AD makes it difficult to diagnose until an acute and catastrophic complication occurs [10]. Second, the blood pressure (BP) and heart rate (HR) monitoring thresholds recommended by the Guideline of the European Society of Cardiology (hereafter *the Guideline*) are insufficient for capturing the dynamic characteristics of BP and HR that are highly associated with the varied and complex nature of AD [11, 12]. Third, population-based regulation fails to provide individualized treatment for patients with different features [13, 14].

Our novel statistical methods offer practitioners fresh prospects for the prescreening and in-hospital treatment of AD. Concretely, we begin by addressing the prescreening stage of AD. According to *the Guideline*, prophylactic interventions for preventing AD should be recommended when the ascending aortic diameter reaches 5.5 cm [15]. However, many mainstream researchers disagree with this recommendation, as it is based on evidence from aortic aneurysm patients instead of AD patients [16]. More importantly, studies have shown that an increase in aortic diameter is not closely associated with the occurrence of type B AD (TBAD) – many patients who experience TBAD have normal aortic diameters [17–21]. Since aortic morphological deterioration can create abnormal biomechanical changes that result in AD, recent literature has started to use specific attributes such as the centerline length [22–27] or the angle of aortic curvature [28, 29] as predictors. Although these attributes have merits in reflecting deterioration from some aspects, their measurements are sometimes subjective, and certain important risk factors can be overlooked, as AD can be caused by changes beyond arterial elongation or abnormal helical flow in the ascending aorta [26, 30]. For example, Krüger et al. and Heuts et al. used the length of the ascending aorta to predict type A AD (TAAD) and found that length has superior diagnostic accuracy to diameter [22, 27]. However, there is no evidence that this length is sufficient to predict TBAD. Alhafez et al. and Gode et al. predicted AD with the vertex angle of a triangle drawn to connect the apex of the aortic arch and the midlines of the ascending and descending thoracic segments of the aorta [28, 29]. However, the places where the authors drew the triangle were subjective and are challenging to replicate in practice. Additionally, the angle of the aortic curvature cannot reflect other geometric changes, such as the elongation and tortuosity of the dissected aorta.

Inspired by clinical evidence demonstrating that AD is mostly accompanied by elastin breakdown and fracture of the aorta, we introduce *the degree of slumpness* to comprehensively capture the morphological changes of the vessel. To the best of our knowledge, we are the first to propose a predictive model of AD that merges pure data-driven inference and expert perceptions (i.e., the abnormal changes in geometry often observed for dissected aortas).

Assuming that AD has occurred, we then focus on the in-hospital treatment stage. Similar to the aforementioned prophylactic intervention, the recommendation from *the Guideline* (i.e., lowering AD patients’ systolic BP (SBP) to 100-120 mmHg and the HR to 60 bpm [10, 31]) is also based on facts from other medical fields and is not very effective in AD clinical practice [13, 32], mainly due to the following reasons. First, the momentary thresholds recommended here ignore the highly dynamic nature of BP and HR, which plays a vital role in triggering cardiovascular events [33, 34]. Second, population-based control thresholds fail to provide individualized BP and HR control for patients with different physical and clinical features. Thus, understanding that adverse events^1^ (AEs) are highly related to wall stress changes on the diseased segment of the aorta, we construct a predictive model using functional data analysis (FDA) to find the optimal timing for BP and HR interventions that minimizes the likelihood of producing an AE.

## Methods

### Morphology-based prediction of AD using the aortic centerline

This prescreening study of AD was retrospective, multicenter, and cross-sectional. We collected the CT scans of consecutive AD patients who underwent thin-cut (0.6-mm) contrast-enhanced CT angiography (CTA) and of healthy individuals who underwent thin-cut CTA or contrast-enhanced chest CT at two institutions (Shanghai Ninth People’s Hospital Affiliated with Shanghai Jiao Tong University School of Medicine and the Vascular Department of the First Affiliated Hospital of Anhui Medical University) between January 2017 and December 2018.

**Fig. 1:**
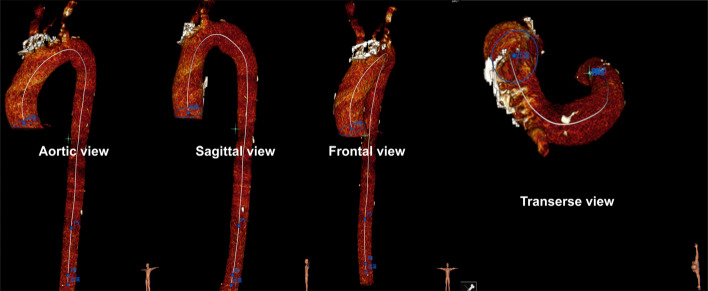
The shape of the aorta viewed from different directions. The shape of the aorta varies when viewed from different perspectives. Aortic view (i.e., the left-anterior oblique 45-degree view for most patients) is recognized as the most accurate direction to observe aorta in clinical practice

**Fig. 2:**
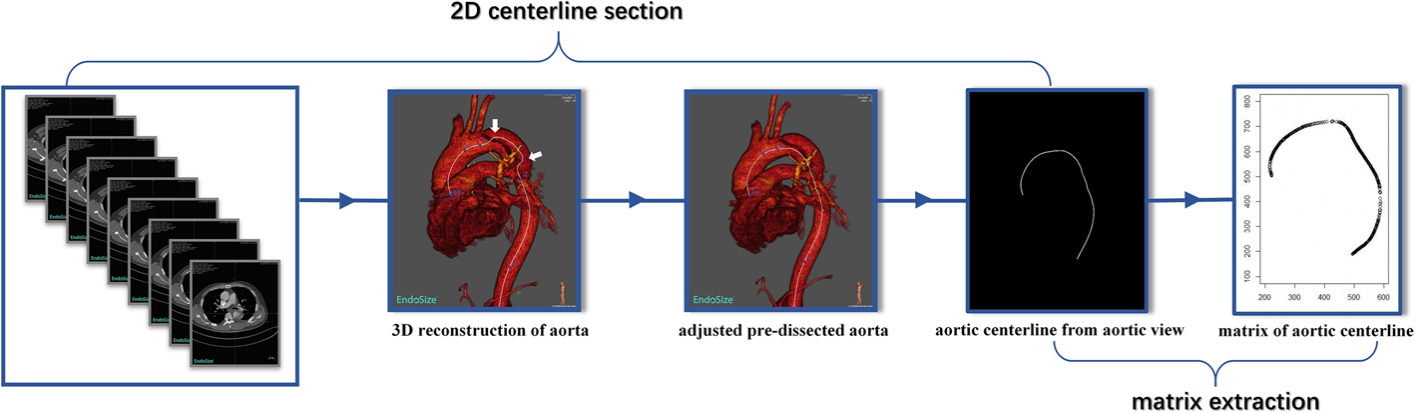
Data processing procedure. The data processing procedure of the 2D centerline includes two sections. First, we restored the pre-dissected centerline from the CTA and projected the 3D centerline down to a 2D centerline. Next, each graph was represented as a matrix

To discern the differences in the shapes between healthy and dissected centerlines, we first restored the predissected centerline from the observed centerline^2^ given the relative stability of the aortic centerline^3^ before and after the onset of AD [26, 43]. Then, based on the principle of parsimony in statistical modeling, the 3D centerline is projected down to 2D from its aortic view (i.e., the left-anterior oblique 45-degree view for most patients), which still sufficiently reveals the aortic morphology. Specifically, while the shape of the aorta varies when viewed from different perspectives, as shown in Fig. 1, the aortic view provides the largest unfolding shape and is the easiest in which to observe a morphological change. In fact, the aortic view has been recognized as the most accurate direction for observing the 2D centerlines in surgical planning and has been widely applied in daily clinical practice [44, 45]. Moreover, compared with 3D centerlines, which require high-priced reconstruction and analysis tools^4^, 2D centerlines can be easily drawn using existing standard CT reconstruction software with little additional cost and thus are accessible to a much larger population. The detailed patient inclusion and exclusion criteria are discussed in Appendix A.1.1 in Additional file 1, and the data processing procedure is visualized in Fig. 2.

**Fig. 3:**
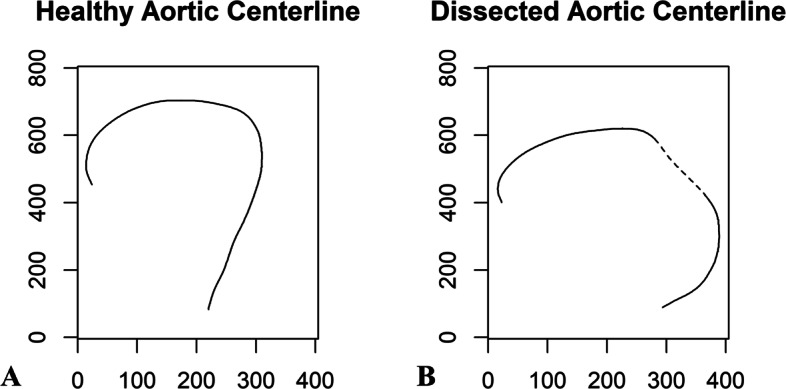
Comparison between healthy and dissected aortic centerlines (example). Illustrations of *the degree of slumpness* of the healthy and dissected aortic centerlines. As shown, certain segments of the dissected centerline become more flat and more slumped

Next, we numerically derived the slope curves from the 2D centerlines^5^ to separate the healthy aortas from the dissected aortas. Four statistics–the average of the slopes, the average of the absolute values of the slopes, the squared values of the slopes, and the aortic tortuosity–were calibrated to characterize *the degree of slumpness*. These metrics together provide a more generalized description of the aortic geometry than the preceding approaches. Particularly, the first three statistics measure the central tendency of the direction and the steepness of the centerline [46–48], which reflect the elastin content of the aorta [49]. Tortuosity captures the irregular twists of the centerline [50–53] and reveals whether the vessel is likely to be predisposed to hemodynamic damage [25]. Indeed, different from healthy aortic arches, which are rounded, dissected arches normally appear to be slumped from various directions, resulting in acute angles at different positions. Figure 3 presents a typical conformation for dissected aortas that was initially introduced by Ou et al., the gothic shape, where the aortic arch slumps from the upper right (i.e., the dotted region in the figure), resulting in acute angulation between the ascending and descending segments [49]. Because of the slumpness of the aorta, certain segments of the dissected centerline become flatter, causing the first three statistics to be smaller than those of the healthy centerline. Additionally, the slumped aorta is expected to be more tortuous due to its elongation and asymmetrical flow profile. The detailed attribute selection process and the approximation measures are presented in Appendix A.2.1 in Additional file 1.

All variables are expressed as the mean ± standard deviation, and the two groups were compared based on the two-sample Kolmogorov-Smirnov test. A two-tailed *p*-value <0.05 implies that the statistics of the healthy and AD individuals are statistically significantly different. Moreover, to ensure comparability between our findings and conventional results, we fed these four statistics into a multivariable model^6^ and assessed their discriminative performance with the area under the receiver operating characteristic curve (AUC), the true positive rate (TPR), and the area under the precision-recall curve (AUPRC). The threshold used to determine the TPR is that which yields the highest sum of sensitivity and specificity, and the baseline AUPRC is the ratio of positives (i.e., the number of AD patients) to the total sample size.

### Dynamic regression analysis of adverse events of AD using FDA

This study of AD treatment was retrospective, multicenter, and cross-sectional. Clinical data were obtained from AD patients from the two aforementioned institutions. Each sample included BP and HR data from up to 14 days since all AD patients were either prepared for emergency surgery after admission or monitored for up to 14 days, when the flap thickness and the growth of the aortic diameter stabilized. We elaborate on the detailed patient inclusion and exclusion criteria and the definition and collection of clinical data in Appendix A.1.2 in Additional file 1.

Here, we first examined the need to incorporate FDA to estimate the BP and HR processes. Previous studies have cautioned us that when the underlying process is continuous, there is no guarantee that it can be consistently estimated from the analysis based only on its discrete counterpart [59–62]. Thus, continuous generalization is an essential prerequisite when dealing with discrete time-series observations, including the BP and HR processes in our context. In addition, many frequently used parametric models for data indexed by time do not admit the temporal aggregation property such that their continuous generalization can lead to an indefinite number of different limits [63–65], and failure in identifying the true limit can result in misspecification. Therefore, we adopted the FDA approach^7^, which has been shown to automatically adapt to the correct limit and recover the true underlying structure from discretely observed data [73–79].

We then used the estimated processes to model the association of BP and HR patterns with the AE rate using the fitting functional generalized linear model (FFGLM). Specifically, we used the patient outcome, which is either stable or experiencing AEs, as the dependent variable; the independent variables included the functional BP and HR processes and the non-functional clinical characteristics. This regression allowed us to estimate the patient’s probability of encountering an upcoming AE based on the observed physiological, physical, and clinical statistics. The complete estimation process is illustrated in Appendix A.2.2 in Additional file 1.

For presentation purposes, we fixed all other variables at their means^8^ and demonstrated the average marginal effects (AMEs) of unit increases in SBP, diastolic BP (DBP), and HR on the AE rate. Two-tailed *p*-values <0.05 were considered to be statistically significant. We compared the discriminative capability of our FFGLM and a traditional linear model^9^ through the AUC, TPR, and AUPRC, where the baseline AUPRC equals the fraction of positives (i.e., patients experiencing AEs) in the dataset. Similar to the FFGLM model, the traditional linear model used the patient outcome as the dependent variable, while its independent variables included all the non-functional clinical characteristics and the shock variable^10^, which indicates whether a patient’s SBP is less than 80 *mmHg* with organ hypoperfusion unresponsive to resuscitative methods or his/her HR is greater than 100 *bpm* [54–56, 58].

## Results

### Morphology-based prediction of AD using the aortic centerline

The AD group included 348 patients, and the healthy control group included 171 individuals. Table 1 shows that the average of the slopes, the average of absolute values of the slopes and the squared values of the slopes for the AD group are all significantly lower than those for the healthy control group, while the tortuosity is significantly higher for the AD group. The AUC for our model was 0.743, and the TPR was 0.865 with a threshold of 0.580, implying that 86.5*%* of the AD patients in our sample can be correctly identified at the chosen threshold. The AUPRC was 0.844, which is noticeably higher than the baseline AUPRC of 0.671.

**Table 1:** Comparison between AD and control groups

	AD group	Healthy control group	*p*-values
Average of Slopes	0.74 ±0.72	1.42 ±1.05	<0.001
Average of Absolute Values of Slopes	2.82 ±0.63	3.37 ±1.09	<0.001
Squared Values of Slopes	29.63 ±21.16	41.28 ±36.26	<0.001
Aortic Tortuosity	2.69 ±0.60	2.27 ±0.68	<0.001

### Dynamic regression analysis of adverse events of AD using FDA

**Fig. 4:**
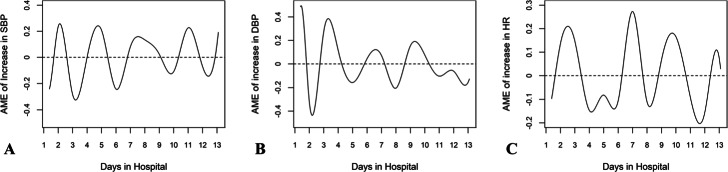
AME of increases in SBP; DBP; HR on changes in AE rate. **a** AME of increases in the SBP on changes in the AE rate. **b** AME of increases in the DBP on changes in the AE rate. **c** AME of increases in the HR on changes in the AE rate. AME = average marginal effect; BP = blood pressure; HR = heart rate; SBP = symbolic BP; DBP = diastolic BP; AE = adverse event

**Fig. 5:**
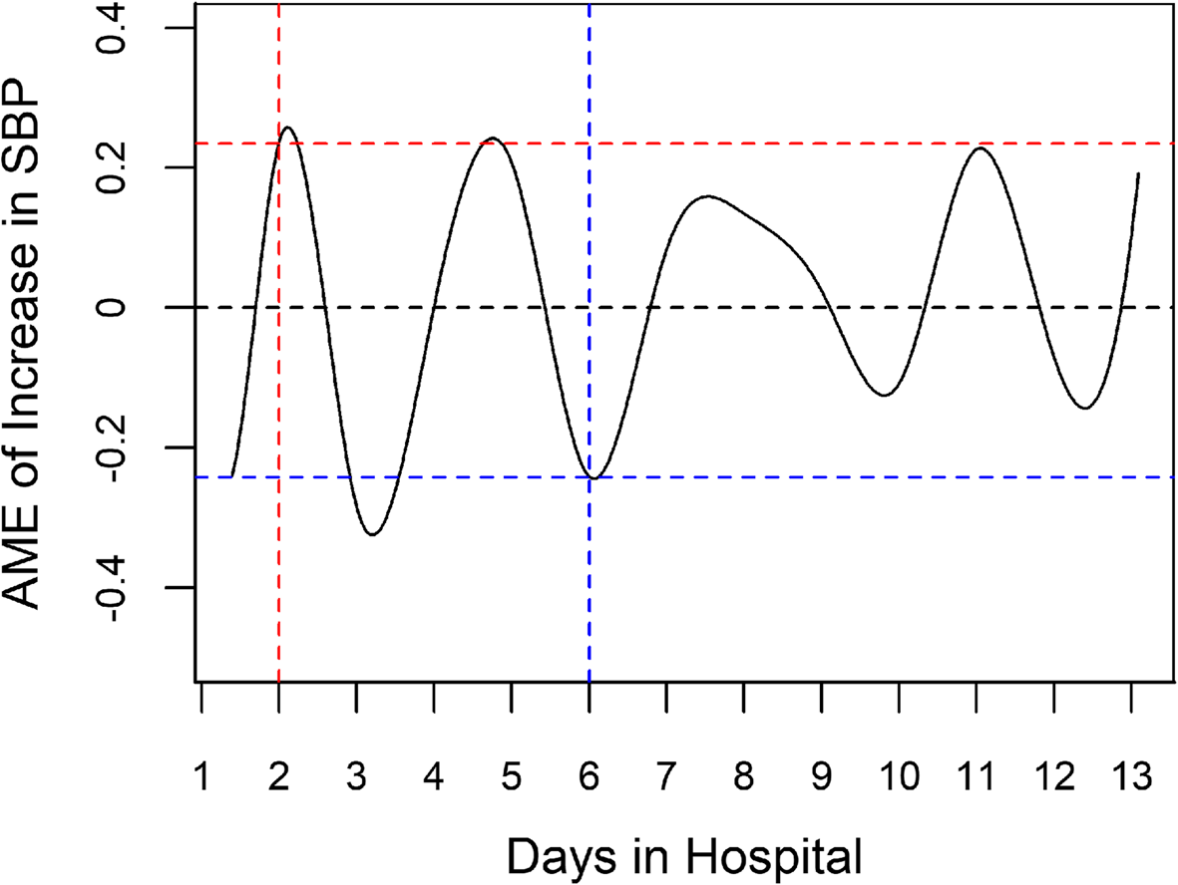
AME of increases in the SBP on changes in AE rate (quantitative analysis). The figure presents the AME of increases in the SBP on changes in the AE rate. AME = average marginal effect; SBP = symbolic blood pressure; AE = adverse event

A total of 458 AD patients were included in the second study, among whom 120 experienced AEs and 338 remained stable. The AMEs of unit increases in SBP, DBP, and HR on the AE rate are illustrated in Fig. 4. Taking Fig. 4a as an example, the curve describes the AME of a unit increase in the SBP on the probability of experiencing an AE at different time points. Specifically, the number of days the patient stayed in the hospital is represented on the x-axis, and the AME is represented on the y-axis. The detailed derivation of the AME is discussed in Appendix A.2.2 in Additional file 1. A positive AME implies that an increase in the SBP increases the likelihood of an AE, at which preventive interventions to control SBP improve the patient survival rate. In contrast, a negative AME suggests that an increase in SBP decreases the likelihood of an AE, at which further SBP controls are dispensable. Specifically, as illustrated in Fig. 5, on day two at midnight, a unit increase in the SBP, on average, increases the probability of encountering an AE by 23.5*%*, implying that the practitioners need to better control the patient’s BP. In contrast, on day six at midnight, an increase in SBP, on average, decreases the probability of encountering an AE by 24.2*%*, implying a reduction in the impact of aortic shear stress on AD progression, and no SBP intervention is required. Indeed, all three AME patterns cyclically fluctuate around 0, and more volatile fluctuations are observed during the first seven days than during the last seven days, especially for DBP, which is consistent with the natural evolution of the dissected aorta.

Table 2 summarizes the AMEs of the change in non-functional determinants on the change in the AE rate. Our results imply that treatments to modify controllable risk factors, including complicated AD, shock, Marfan syndrome, aortic diameter, and pericardial effusion, can significantly increase patient survival rates and should be implemented if possible. For instance, patients with Marfan syndrome, on average, have a higher probability of experiencing AEs, 21%, than other AD patients. The AUC and TPR of our model were 0.839 and 0.808 (with a threshold of 0.223), respectively, both of which were larger than those of the traditional linear model, 0.797 and 0.725 (with a threshold of 0.270). The baseline AUPRC was 0.299, while that of our model was 0.668, and that of the traditional model was 0.601.

**Table 2:** AMEs of clinical characteristics on AE rate

Factor	AME	*p*-values	95% C.I.
Age	-0.00	0.65	(-0.00, 0.00)
Male sex	0.11	<0.001	(0.01, 0.20)
Marfan syndrome	0.21	0.01	(0.05, 0.37)
Time from onset to admission	-0.00	0.18	(-0.00, 0.00)
Family history of aortic disease	-0.15	0.12	(-0.33, -0.01)
History of diabetes mellitus	0.04	0.65	(-0.13, 0.20)
History of hypertension	-0.01	0.87	(-0.08, 0.07)
History of cardiovascular disease	0.03	0.51	(-0.06, 0.12)
Chronic renal insufficiency	-0.16	0.04	(-0.32, -0.01)
Stanford type B AD	-0.14	0.01	(-0.24, 0.04)
Complicated AD	0.12	<0.001	(0.04, 0.20)
Shock	0.13	<0.001	(0.04, 0.22)
Abdominal vessel involvement	0.12	<0.001	(0.04, 0.20)
Maximum aortic diameter ≥5.5 cm	0.09	0.04	(0.00, 0.18)
Arch vessel involvement	0.13	<0.001	(0.05, 0.21)
Pericardial effusion	0.15	0.01	(0.04, 0.25)
Pleural effusion	-0.04	0.35	(-0.14, 0.05)

## Discussion

The first finding of this study demonstrates significant differences in aortic shapes between healthy and AD patients. The lower values of the average of the slopes, the average of the absolute values of the slopes, and the squared values of the slopes for the AD group are consistent with the pathological changes of elastin reduction in AD. The higher value of tortuosity for the AD group coincides with the theory that increased tortuosity is likely to predispose the vessel to hemodynamic damage [25]. Altogether, these results indicate that the dissected aortic centerlines tend to be more slumped than the healthy centerlines, which matches the observations in clinical practice.

The second finding reveals that variations in BP and HR have a strong predictive power for AEs. All three AME patterns are consistent with the J-shaped relationship between BP and AEs found in clinical practice; the highest mortality rates are observed for patients with SBP ≤ 100 mmHg or >180 mmHg [55], which confirms again that increases in BP and HR are sometimes desirable. More specifically, during certain stages of AD, a rise in BP can lead to further tearing of the aortic wall, resulting in severe aortic rupture [11, 83]. In contrast, when the patient’s BP is relatively low, a further decrease in BP can lead to cerebral or visceral ischemia, which is the most common cause of AEs in AD [84, 85]. Different from *the Guideline* recommendation, our algorithm allows for natural fluctuations in BP and HR, and interventions are only advised when increases in the rate of AEs are detected. Thus, with less antihypertensive medication, the potential side effects from the intake of those drugs can be reduced [13]. Additionally, the less volatile fluctuations during the last seven days coincide with the natural evolution of the dissected aorta; the impact of aortic wall stress on the progression of AD decreases in the natural transition from the acute to the chronic stage [86]. Moreover, since our model can predict the occurrence of AE in advance, treatment can be implemented a priori to avert the irreversible damage induced by sudden increases in BP or HR [87]. In summary, our model provides practitioners with guidance on the optimal timing for preventive interventions for BP and HR to offer the highest survival rate.

A limitation of the present study is that the retrospective and observational nature of the two investigations may have led to bias. In theory, the data should be collected from a diverse group of volunteers, each with aortic CT records obtained over an extended period and among them, some developed AD. However, due to the low incidence of AD, such data are exceptionally rare in practice. To date, the most extensive dataset of this kind includes 17 predissected CT scans out of the 579 observations and is used to assess the role of aortic elongation in the prediction of TAAD [88]. Since our model adopts more complicated metrics to investigate the risk factors for AD and deals with infinite dimensions, such a small sample size is not adequate for analysis. It is also noteworthy that our purpose was to discern the shape difference between the healthy and dissected centerlines regardless of the initial factors, including but not limited to aging and genetic history; thus, the direct comparison of the healthy and AD individuals is sensible in the current context.

In addition, since both analyses are of infinite dimensions, the sample size was relatively small. With a greater amount of available data, our estimators will converge to their true values, and hence their predictive power will improve.

## Conclusion

In this paper, we adopt novel statistical methods to shed light on the prevention and treatment of AD, a fatal disease known for ages. Different from previous approaches that can only address aortic deterioration from specific aspects, we introduce the term *degree of slumpness* to depict aortic morphological changes comprehensively. Thus, practitioners can extract more information from CT scans at a lower cost for a larger population so that patients with a tendency to suffer from AD can be detected in advance. Hence, prophylactic treatment can be administered a priori to mitigate potential risk factors.

Moreover, our paper contributes to knowledge on the treatment stages of AD. The dynamic model reveals that BP and HR variations have a strong predictive power for AEs. This finding suggests that controlling patients’ BP and HR according to the likelihood of the AE occurrence may provide the patient with the highest possible survival probability.

We are the first to incorporate image recognition techniques and FDA in predicting the occurrence of AD and its AEs. This full set of studies of AD can provide practitioners with insights into both its prescreening and real-time treatment. With further clinical trials, our system has the potential to be operated without the presence of experts.

## Supplementary Information

**Additional file 1** Appendix A: Data and Methodologies

## Data Availability

The datasets analyzed during the current study are not publicly available due to the highly sensitive nature of medical records but are available from the corresponding author on reasonable request. Declarations

## Abbreviations

AD: Aortic dissection
AE: Adverse event
AME: Average marginal effect
AUC: Area under the receiver operating characteristic curve
AUPRC: Area under the precision-recall curve
BP: Blood pressure
CTA: Contrast-enhanced CT angiography
DBP: Diastolic BP
FDA: Functional data analysis
FFGLM: Fitting functional generalized linear model
HR: Heart rate
SBP: Systolic blood pressure
TPR: True positive rate

## Footnotes

1 We classify patients as experiencing an adverse event if any of the following progressive events of AD occur during medical management: (1) fatal or nonfatal aortic rupture; (2) organ or limb ischemia; (3) aortic aneurysm formation (defined as a maximum thoracic aortic diameter ≥6 cm); (4) rapid aortic growth (defined as a diameter increase of ≥10 mm within one year); and (5) death.

2 The detailed restoration method is presented in Appendix A.1 in Additional file 1.

3 The centerline morphology has been widely used in medical studies to predict disease [35–37]. In addition, because measurement of the centerline is a necessary step for surgical protocols in clinical practice, current centerline extraction methods are readily available and reliable [38–42]. Nevertheless, if the centerline approach does not work well, one might try other machine learning methods, such as a convolutional neural network, which are currently popular in medical studies. These methods may be more data-driven and capable of predicting AD using aortic images directly than centerline measurement. However, a discussion of those approaches is beyond this paper’s scope, and we leave it to future research.

4 Mimics software with 3D measurement tools costs more than <DOLLAR/>12,000 with a maintenance package of <DOLLAR/>900 per year.

5 Here, we define the slope curve as the collection of slopes of the tangent lines at each point on the centerline.

6 The multivariable model used here is the logistic model, which is widely applied to assess the discriminative capability of the risk factors associated with AD [54–58]

7 FDA has been widely applied in a large number of disciplines, such as biomedical science, medicine, economics, finance, linguistics, psychology and sports [66–72]

8 Different sets of figures can be produced with the controlled variables fixed at other values.

9 Most studies predicting the probability of AEs in AD used the traditional linear model [11, 54–56, 58, 80–82], and we are not aware of other state-of-the-art methods in this field.

10 The use of shock is the most extreme case in incorporating BP and HR in AE predictions and thus provides the strongest predictive power. Mainly, a patient is in shock when his/her BP and HR are such that his/her condition is no longer reversible, and subsequent aggressive interventions can lead to serious complications.
