# Supplementary material for: Prescreening and treatment of aortic dissection through an analysis of infinite-dimension data
Source: BioData Min. 2021 Apr 1;14:24. doi: 10.1186/s13040-021-00249-8 (PMC8015064; doi:10.1186/s13040-021-00249-8)
Supplement: Supplementary file 1 — Additional file 1 Appendix A: Data and Methodologies [file 13040_2021_249_MOESM1_ESM.pdf]

Appendix A: Data and Methodologies

A.1 Data

In this subsection, we present the detailed data collection processes for the two prediction models.

A.1.1 Morphology-based prediction of AD using the aortic centerline

The data used to predict AD occurrence were retrospectively collected from the local aortic registry database. We retrospectively evaluated CT scans of consecutive AD patients who underwent thin-cut (0.6 mm) contrast-enhanced CT angiography between January 2017 and December 2018. The exclusion criteria for the AD group were as follows: (1) patients with connective tissue diseases (i.e., Marfan's disease, Loeys-Dietz syndrome or Ehlers-Danlos syndrome); (2) patients with iatrogenic dissection; (3) patients with other aortic diseases such as aneurysms; (4) patients with previous aortic surgeries; (5) patients with previous cardiothoracic diseases or cardiothoracic surgeries; (6) patients with diseases that might distort thoracic aortic morphology (i.e., pulmonary nodules with diameter $> 3\ cm$, mediastinal masses or lymph nodes with diameter $> 1\ cm$, pneumothorax, pulmonary bullae with diameter $> 3\ cm$, history of thoracic or mediastinal surgeries, etc.); and (7) patients with diseases that might distort the shape of the thoracic wall (i.e., scoliosis, barrel chest, pectus carinatum, history of spinal surgeries, etc.).

We retrospectively evaluated CT scans of consecutive patients with healthy aortas who underwent thin-cut (0.6-mm) contrast-enhanced CT angiography or contrast-enhanced chest CT between April 2018 and December 2018 as a healthy control group. The exclusion criteria for the healthy control group were as follows: (1) patients with suspected or known aortic diseases; (2) patients with previous cardiothoracic diseases or cardiothoracic surgeries; (3) patients with diseases that might distort thoracic aortic morphology; and (4) patients with diseases that might distort the shape of the thoracic wall.

The procedures follow STROBE guidelines and the Declaration of Helsinki, and the study protocol was reviewed and approved by the local ethics committee. The need for written patient consent was waived because of the observational nature of this study. This retrospective factorial study was registered with the Chinese Clinical Trial Registry, and its registration number is ChiCTR2000029219.

A.1.2 Dynamic regression analysis of aortic adverse event using conditioning on functional covariates

The data used in the predictive model for AEs have retrospectively reviewed adult inpatients with Type-A and Type-B non-traumatic AD between January 2004 and December 2018. Clinical evaluations confirmed the diagnosis based on imaging modalities such as contrast-enhanced computed tomography angiography (CTA) and Magnetic Resonance Imaging.

Type-A AD and complicated Type-B patients were monitored for BP and HR while preparing for emergency surgery upon arrival at the hospital, and uncomplicated Type-B AD patients were monitored for BP and HR during medical management until the occurrence of AE or up to 14 days (i.e., the subacute phase). BP and HR were measured every 5 minutes to 2 hours, depending on the condition of the patient using automated non-invasive BP monitors.

Predictors are selected based on established risk factors related to AEs of AD and potential factors reported in previous studies. Clinical data are reviewed from the electronic medical records including demographics (i.e., age, sex, Stanford type of AD), clinical presentation, medical history (i.e., Marfan syndrome, family history of aortic disease, history of hypertension, history of cardiovascular diseases, history of diabetes mellitus, chronic renal insufficiency), and outcomes. Imaging data of the aorta (i.e., arch vessel involvement, abdominal vessel involvement, maximum aortic diameter ≥ 5.5 cm, pericardial effusion, and pleural effusion) are obtained from CTA or electronic records. In this paper, complicated AD is defined as persistent pain, uncontrolled hypertension, early aortic expansion, malperfusion, and signs of rupture. Hypotension is when the SBP is less than 90 $mmHg$, and shock is when SBP is less than 80 $mmHg$ with organ hypoperfusion not responsive to resuscitative methods. To predict AEs and interfere with BP and HR before irreversible circulatory imbalance occurs, we deleted observations with SBP less than 40 $mmHg$ (i.e., half of the IRAD-defined shock threshold).

The procedures follow the TRIPOD statement, and the study protocol is reviewed and approved by the local ethics committee. The need for written patient consent is waived because of the observational nature of this study. This retrospective factorial study was registered with the Chinese Clinical Trial Registry, and its registration number is ChiCTR1900025818.

A.2 Statistical Methods

In this subsection, we discuss the detailed statistical methods applied in predicting the likelihood of AD and its AEs.

A.2.1 Morphology-based prediction of AD using the aortic centerline

Digital Imaging and Communications in Medicine standard data from eligible patients were extracted for analysis anonymously. The 3D multi-planar reconstruction of the aorta was semi-automatically created using EndoSize software version 3.1 (Rennes, France). A centerline was created from the sinotubular junction to the diaphragmatic level of the descending thoracic aorta. The following procedures were followed to obtain the morphological details of the pre-dissected aorta. Since the diameter of the aorta increased by about 30% after the dissection, and the centerline remains unchanged, we only retain the aortic centerline [1, 2]. In the presence of AD, we manually place the seed points of the centerline at the center of the total artery lumen (i.e., both the true and false lumens) to obtain the adjusted pre-dissected aortic centerline.

The 2D image of the aortic centerline was measured in the aortic view. Each sample is a graph where points on the centerline are marked as 1 representing white color, and others are marked as 0. Thus, each graph can be represented as a matrix. The detail of recording a centerline for each graphic files are as follows: (1) read the AD image data from its graphics file; (2) convert the true color RGB image to the gray-scale image ranged from 1 to 255; (3) convert the gray-scale image into a matrix, for each element in the matrix, if the gray-scale is greater than 90, then save it as 1 to represent white color; otherwise, save it as 0 to represent black color; (4) represent the matrix as x-axis and y-axis, where the left bottom position of the matrix represents $(1, 1)$; (5) iterate from bottom to up on the left half of the matrix, and then from up to bottom on the right half of the matrix, for each consecutive 1's (white cell's) on the matrix, only record its middle position (i.e., the x-axis and y-axis values) on the matrix; and (6) return the saved positions according to Step 5, which gives the centerline of an AD image, where the first point represents the starting position, and the last point represents the ending position. As shown in Figure 1, the aortic centerline of a patient with AD is more slumped than that of a healthy person.

Figure 1 Illustration of the Tangent Curves for Healthy and Dissected Aortic Centerlines

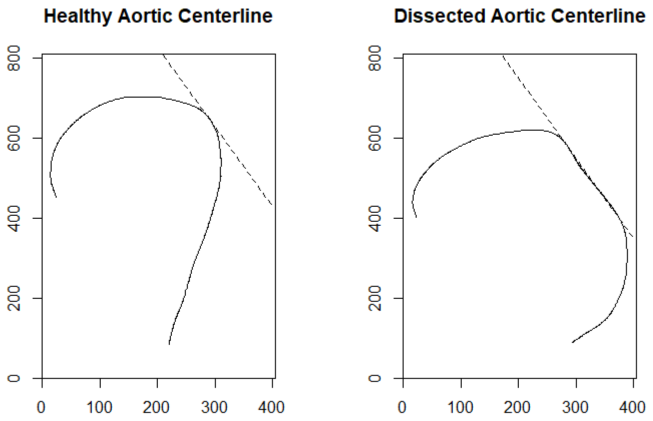

The dotted line on each demonstration in Figure 1 represents the tangent curve at a point of the centerline. We measure the slope of each tangent curve ($\rho(\cdot)$) as the Euclidean distance between the two closet points. That is,

$$\rho(A, B) = \sqrt{(A_x - B_x)^2 + (A_y - B_y)^2},$$

where A and B are two consecutive points on the centerline. The length of the centerline (L) is the summation of slopes of all tangent curves, and D is the Euclidean distance between the start and the end points. The tortuosity of the centerline (T) is represented by the length of the centerline divided by the Euclidean distance between the start and the end points (i.e., $T = \frac{L}{D}$). The two-sample Kolmogorov–Smirnov test is utilized to test whether the the aortic centerline of the AD group is more slumped than the healthy control group.

For the robustness check, we measure the tangent curves at each point by taking the average of the slopes between the point and its closest points, excluding the start and the end points. As shown in Table 1, the trends for all four attributes are consistent.

Table 1 Comparison between AD and Control Groups — Robustness Test

	AD group	Healthy control group	p-values
Average of slope	0.74 ± 0.71	1.42 ± 1.04	< 0.001
Average of absolute slope	2.72 ± 0.59	3.25 ± 1.01	< 0.001
Squared slope	19.89 ± 13.04	27.16 ± 21.09	0.001
Aortic tortuosity	2.69 ± 0.60	2.27 ± 0.68	< 0.001

A.2.2 Dynamic regression analysis of aortic adverse event using conditioning on functional covariates

Let $X_{i,v,t}$ be the value of X_v at time t for patient i, where X_v is the discrete observations of v and $v \in (SBP, DBP, HR)$. Assuming the smoothness in the underlying process of X, denoted $W_{i,v}(t)$, we express $X_{i,v,t}$ as

$$X_{i,v,t} = W_{i,v}(t) + \epsilon_{i,v}(t),$$

where $\epsilon_{i,v}(t)$ is a random error with mean zero and finite variance.

First, we adopt FDA to capture patients' underlying BP and HR processes. Based on the standard leave-one-out cross-validation (CV) method [3], we select the optimal number of basis functions (H) to be 107 and the optimal smoothing parameters (γ_v) to be 1.29155×10^{-9}. Therefore, $W_{i,v}(t)$ can be approximated by

$$W_{i,v}(t) \approx \sum_{h=1}^{H} c_{i,v,h}\phi_{i,v,h}(t),$$

where the coefficients $c_{i,v,h}$ are obtained based on the parameters (H,γ_v) and $\phi_{i,v,h}(t)$ is the basis functions [3].

The FFGLM is an extension of the linear regression models where the conditional expected value, $\mathbb{E}[\cdot]$, is related to the linear prediction η via the logistic link function $g(\cdot)$ as follows:

$$\mathbb{E}\left[Y|\,\mathbb{Z}, \mathbb{W}(t)\right] = \eta = g^{-1}\left(\alpha + \sum_{j=1}^{p}\beta_j Z_j + \sum_{v}\frac{1}{\sqrt{T_v}}\int_0^{T_v} W_v(t)\lambda_v(t)dt\right), \quad (1)$$

where Y denotes the scalar outcome of the patient. $\mathbb{Z} = [Z_1, ..., Z_p]$ is a vector of the non-functional patient characteristics and $\mathbb{W}(t) = [W_{SBP}(t), W_{DBP}(t), W_{HR}(t)]$ is a vector of the functional ones. The value β_j determines the impact of Z_j on the AE occurrence, and the value $\lambda_v(t)$ determines the impact of v at time t on the AE occurrence, where $t \in [0, T_v]$.

We then model the association of changes in BP and HR with the change in the likelihood of AE occurrence using FFGLM [3]. The FFGLM is able to deal with continuous functional covariates with different lengths, which is a frequent problem in practice since patients' hospitalization time varies due to plenty of personal and medical reasons. Further, we interpret the results from FFGLM through the AME.

Note that $\mathbb{E}[\cdot]$ denotes the conditional expectation of the binary outcome, Y, and under the logistic regression setup, it equals the probability of the AE occurrence conditional on all the patient characteristics. Then, the partial derivative of $\mathbb{E}[\cdot]$ with respect to a particular independent variable measures the effect of the change in that variable on the change of the probability of experiencing an AE. Taking SBP as an example, the partial derivative of Equation 1 with respect to $W_{SBP}(t)$ is:

$$\begin{aligned}\frac{\partial\,\mathbb{E}\left[Y\,|\,\mathbb{Z},\mathbb{W}(t)\right]}{\partial W_{SBP}(t)} &= \Lambda'\left(\alpha+\sum_{j=1}^{p}\beta_j Z_j+\sum_v \frac{1}{\sqrt{T_v}}\int_0^{T_v} W_v(t)\lambda_v(t)dt\right)\times\frac{1}{\sqrt{T_v}}\int_0^{T_{SBP}}\lambda_s(t)dt\\ &= \frac{e^d}{(1+e^d)^2}\times\frac{1}{\sqrt{T_{SBP}}}\int_0^{T_{SBP}}\lambda_{SBP}(t)dt,\end{aligned} \tag{2}$$

where $d=\alpha+\sum_{j=1}^{p}\beta_j Z_j+\sum_v\frac{1}{\sqrt{T_v}}\int_0^{T_v}W_v(t)\lambda_v(t)dt$. Then, we substitute each patient's observed SBP sequence into Equation 2 to estimate the marginal effect (ME) pattern during his/her first 14 days in the hospital. Last, the AME curve (i.e., Figure 4A in the paper) is obtained by taking the average of the estimated MEs at each time point.

Author details

References

1. Rylski, B., Muñoz, C., Beyersdorf, F., Siepe, M., Reser, D., Carrel, T., Schoenhoff, F., Schlensak, C., Lescan, M., Eckstein, H.-H., *et al.*: How does descending aorta geometry change when it dissects? European Journal of Cardio-thoracic Surgery **53**(4), 815–821 (2017)
2. Wu, J., Zafar, M.A., Li, Y., Saeyeldin, A., Huang, Y., Zhao, R., Qiu, J., Tanweer, M., Abdelbaky, M., Gryaznov, A., *et al.*: Ascending aortic length and risk of aortic adverse events: the neglected dimension. Journal of the American College of Cardiology **74**(15), 1883–1894 (2019)
3. Ramsay, J.: Functional Data Analysis. Wiley Online Library, ??? (2005)
